# Modulation of Neocortical Development by Early Neuronal Activity: Physiology and Pathophysiology

**DOI:** 10.3389/fncel.2017.00379

**Published:** 2017-11-29

**Authors:** Sergei Kirischuk, Anne Sinning, Oriane Blanquie, Jenq-Wei Yang, Heiko J. Luhmann, Werner Kilb

**Affiliations:** Institute of Physiology, University Medical Center of the Johannes Gutenberg University Mainz, Mainz, Germany

**Keywords:** development, cerebral cortex, subplate, spontaneous activity, somatosensory cortex, rodent, human, review

## Abstract

Animal and human studies revealed that patterned neuronal activity is an inherent feature of developing nervous systems. This review summarizes our current knowledge about the mechanisms generating early electrical activity patterns and their impact on structural and functional development of the cerebral cortex. All neocortical areas display distinct spontaneous and sensory-driven neuronal activity patterns already at early phases of development. At embryonic stages, intermittent spontaneous activity is synchronized within small neuronal networks, becoming more complex with further development. This transition is accompanied by a gradual shift from electrical to chemical synaptic transmission, with a particular role of non-synaptic tonic currents before the onset of phasic synaptic activity. In this review article we first describe functional impacts of classical neurotransmitters (GABA, glutamate) and modulatory systems (e.g., acetylcholine, ACh) on early neuronal activities in the neocortex with special emphasis on electrical synapses, nonsynaptic and synaptic currents. Early neuronal activity influences probably all developmental processes and is crucial for the proper formation of neuronal circuits. In the second part of our review, we illustrate how specific activity patterns might interfere with distinct neurodevelopmental processes like proliferation, migration, axonal and dendritic sprouting, synapse formation and neurotransmitter specification. Finally, we present evidence that transient alterations in neuronal activity during restricted perinatal periods can lead to persistent changes in functional connectivity and therefore might underlie the manifestation of neurological and neuropsychiatric diseases.

## Introduction

Animal and human studies have demonstrated that patterned neuronal activity is an inherent feature of developing neuronal networks. Spontaneous activity patterns demonstrate specific properties during distinct developmental stages, in accordance with the establishment of neuronal connectivity (for review Egorov and Draguhn, 2013; Yang et al., 2016). While at embryonic stages intermittent spontaneous activity is synchronized within small neuronal networks, activity patterns become more complex during further development of the cerebral cortex, depending on the maturational state of network connectivity. Experimental evidence, clinical findings and theoretical considerations demonstrated the importance of neuronal activity for the structural and functional maturation of the neocortex (Thivierge, 2009; Ben-Ari and Spitzer, 2010; Kirkby et al., 2013; Rahkonen et al., 2013). This review aims to summarize our current knowledge about the mechanisms by which specific electrical activity patterns can influence or control structural and functional development. For this purpose, we first describe the development of synaptic transmission and its role in the generation of early neuronal activity patterns in the neocortex. Based on this information, we subsequently summarize how neuronal activity interferes with distinct neurodevelopmental events. Finally, we present evidence that transient alterations in neuronal activity during restricted perinatal periods can lead to persistent changes in functional connectivity and might thereby underlie a variety of neurological and neuropsychiatric disorders.

## Sequence of Neocortical Developmental Events

### Genesis of the Neocortex

**Figure 1:**
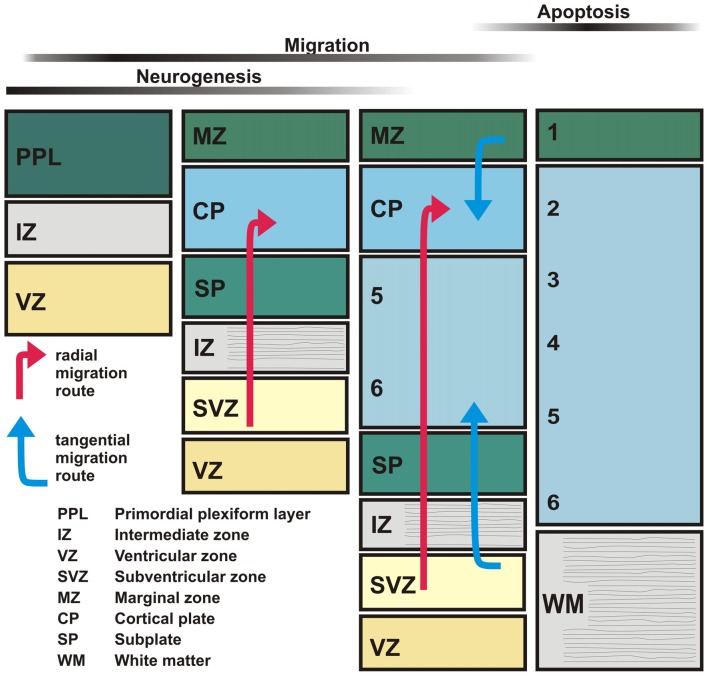
Schematic diagram illustrating the major stages of neocortical development. The earliest-generated neocortical neurons, including Cajal-Retzius and subplate (SP) neurons, form the primordial plexiform layer (PPL). Later-generated neurons generated in the ventricular (VZ) and subventricular zones (SVZ) split the PPL into the marginal zone (MZ) and the SP layer thereby forming the cortical plate (CP). Subsequently newly-generated glutamatergic neurons migrate radially (red arrows) towards the MZ and settle in the CP, thus establishing the inside first-outside last pattern of the developing neocortex. GABAergic interneurons are generated in the ganglionic eminences and reach the neocortex by tangential migration (blue arrows).

Since the developing cerebral cortex shows prominent molecular, anatomical and physiological changes during embryonic and early postnatal stages, we first briefly describe neocortical development and the transient structures occurring during this period (Figure 1). In the mammalian neocortical *anlage*, the earliest generations of postmitotic cells are produced in the dorsal neuroepithelium. These cells migrate towards the outer surface of the pallium and form the primordial plexiform layer (PPL) or preplate (Bystron et al., 2008), which also contains a dense network of monoaminergic and GABAergic fibers (Levitt and Moore, 1979; Dammerman et al., 2000). Subsequently born glutamatergic (mostly pyramidal) neurons are generated in the ventricular (VZ) and subventricular zone (SVZ), germinal layers adjacent to the ventricular surface. Through radial migration these cells form the cortical plate (CP; in rodents at embryonic day [E] 14–17), which divides the preplate into a superficial marginal zone (MZ) and a deeper subplate (SP; for review Frotscher, 1998; Marín-Padilla, 1998). Later born neurons invade the CP and terminate at the upper border of the CP, establishing the inside-first outside-last pattern in neocortical development. Cajal-Retzius cells, a specific population of neurons located in the MZ, are essential for the establishment of this inside-first outside-last pattern (for review Frotscher, 1998; Kirischuk et al., 2014; Kilb and Frotscher, 2016). Early born neurons in the SP are at the same time elementary for the development of adequate thalamocortical connections and the formation of functional cortical columns (for review Luhmann, 2003; Luhmann et al., 2009; Kanold and Luhmann, 2010). Genetic programs control many early developmental events, but neurotransmitters, released constitutively or via activity-dependent processes, are also involved in these events at surprisingly early stages of corticogenesis (for review Owens and Kriegstein, 2002; Luján et al., 2005). Therefore, we will next summarize the maturation of the main neurotransmitter systems during early development of the cortex.

### Maturation of Neurotransmitter Systems

#### GABA

GABA is the major inhibitory neurotransmitter in the adult brain and influences neuronal activity and development at prenatal and postnatal stages (for review Ben-Ari et al., 2007; Kilb, 2011; Luhmann et al., 2014). In rodents, the GABA concentration in the cortical *anlage* at E15 and birth amounts to about 20% and 60% of adult levels, respectively. High levels of GABA in the cerebral *anlage* during prenatal development can be explained by the fact that the GABA transaminase, an enzyme catalyzing the degradation of GABA, is only weakly expressed around birth (Van Eden et al., 1989). GABAergic cells have been found in the plexiform primordium already at E14. At E16 GABAergic cells are found in all fetal zones of the cerebral *anlage* but most of them are located in the MZ and the SP, while at E21 GABAergic cells are also present in the CP.

The expression of vesicular GABA transporters is first observed around birth (Minelli et al., 2003), but evidence for GABAergic synaptic transmission has been reported already in embryonic neocortical neurons (Owens et al., 1999; Kilb et al., 2004). However, other pathways, like volume-sensitive chloride channels (Kimelberg et al., 1990), or reversely operating GABA transporters (Richerson and Wu, 2003) can mediate GABA release, leading to non-synaptic tonic GABAergic activation (for review Kilb et al., 2013). Expression of the neuronal GABA transporter isoform GAT-1, as well the typically glial located isoform GAT-3 has been found in the perinatal rodent brain (Minelli et al., 2003), but during development the expression of these transporters is not restricted to the respective cell type. As GAT reversal potential is suggested to be close to the membrane resting potential (Wu et al., 2006), GAT-mediated GABA release can be induced by neuronal activities either directly from neurons (Wu et al., 2007; but see Savtchenko et al., 2015) or indirectly (by K^+^ elevation or glutamate uptake) from astrocytes (Unichenko et al., 2012).

The expression of GABA_A_ receptors at very early developmental stages was already demonstrated 25 years ago, when GABA_A_ receptor subunits α_3_, α_4_, α_5_, β_2/3_ and γ2 were shown to be expressed in the neocortical *anlage* of E17 rat fetuses (Laurie et al., 1992; for review Luján et al., 2005). Developmental changes in GABA receptor subunits were later confirmed by more recent molecular approaches (e.g., Yu et al., 2006), however these data were limited to pre- and early perinatal stages. Functional GABA_A_ receptors have been described on neuronal precursor cells in the neocortical proliferative zone at E16 (LoTurco et al., 1995). At E18 GABA_A_ receptors, identified by benzodiazepine-binding, are mainly located in the MZ and the SP, while around birth GABA receptors are expressed throughout the CP with an inside-out density gradient (Schlumpf et al., 1983; for review Galanopoulou, 2008). In contrast, functional GABA_C_ receptors have been observed in the neocortex only on putative migrating neurons in the intermediate zone (Denter et al., 2010). As ionotropic GABA_A/C_ receptors are directly linked to a chloride conductance and the intracellular chloride concentration is shifted to higher values during development (Yamada et al., 2004), these receptors mediate a depolarizing, and sometimes excitatory action during prenatal and early postnatal development (Ben-Ari et al., 1989; Leinekugel et al., 1997; for review Ben-Ari et al., 2012). Recent experiments demonstrated depolarizing, but inhibitory effects of GABA_A_ receptors in the developing neocortex under *in vivo* conditions under anesthesia (Kirmse et al., 2015) and that photostimulation of GABAergic neurons *in vivo* attenuates neuronal activity in the neocortex and hippocampus of immature mice (Valeeva et al., 2016). However, further experimental studies are required in different neuronal populations, under non-anesthetized conditions and with physiological monosynaptic GABAergic activation to understand the role of GABA action in developing cortex (for discussion of this issue see Ben-Ari, 2015). In addition to the ionotropic GABA_A/C_ receptors, metabotropic GABA_B_ receptors have been found in the neocortex at E14 and are expressed in Cajal-Retzius as well as in SP neurons (López-Bendito et al., 2002).

#### Glutamate

Glutamate, the major excitatory neurotransmitter in the adult mammalian brain mediates important trophic roles in development including proliferation, migration and neuronal survival (for review Lipton and Kater, 1989; Owens and Kriegstein, 2002). The expression of glutaminase, the main enzyme required for glutamate synthesis, shows a rapid increase at E17 soon after the glutamatergic projection neurons enter the CP in rodents (Larsson et al., 1985, compare to http://developingmouse.brain-map.org/gene/show/14436). Vesicular glutamate transporters, which are essential elements for synaptic glutamate release, are expressed in the neocortical *anlage* of rodents already at E14 (Schuurmans et al., 2004). Glutamate can also be released via nonsynaptic mechanisms. Although the excitatory amino acid transporter (EAAT) 1 and EAAT3 are expressed in the developing neocortex at late embryonic and early postnatal stages (Sutherland et al., 1996; Furuta et al., 1997), these glutamate transporters cannot be reversed under physiological conditions and thus most probably do not contribute to nonsynaptic glutamate release (Levy et al., 1998). Possible candidates mediating nonsynaptic glutamate release upon neuronal activity are gap junction hemichannels (Ye et al., 2003) or ATP receptors (Duan et al., 2003), but their contribution to glutamate release in the perinatal developing cortex has to be elucidated.

The expression of both major subtypes of ionotropic glutamate receptors, AMPA/kainate and NMDA receptors, has been identified by *in situ* hybridization in the prenatal neocortex. In mice, the NMDA receptor subunits NR1 and NR2B are expressed in the neocortical *anlage* already at E17, while NR2A expression appears around the end of the first postnatal week (Monyer et al., 1994; for review Luján et al., 2005), a developmental expression pattern that has recently been confirmed in human fetal tissue (Bagasrawala et al., 2017). The AMPA/kainate receptor subunits GluR1, GluR2 and GluR4 are also expressed in the neocortical *anlage* already at E14 (Monyer et al., 1991; for review Luján et al., 2005). Cells in the VZ have been shown to respond to application of glutamate already at E15–16. As NMDA fails to induce any detectable response at this age, neuron precursor cells in the neocortical proliferative zone most probably only express functional AMPA receptors until E16 (LoTurco et al., 1995). However, the observation that between E16 and E18 NMDA agonists and antagonists modulate neuronal migration (Hirai et al., 1999; Kihara et al., 2002) implies that at least migrating neocortical neurons express functional NMDA receptors during this developmental period.

#### Glycine

Glycine is an inhibitory neurotransmitter located predominantly in the spinal cord, but it is also important for neocortical development. *In situ* hybridization and immunohistochemical experiments revealed that α_2_ and β subunits of glycine receptors are present in the superficial layers of the developing cerebral cortex at embryonic stages (Becker et al., 1993; Avila et al., 2013). In accordance with this data, in embryonic and early postnatal neocortical neurons electrophysiological experiments revealed functional glycine receptors, which are most probably α_2_/β heterodimeric receptors (Flint et al., 1998; Okabe et al., 2004). Since glycine receptors open ligand-gated Cl^−^ channels, activation of glycine receptors leads to a depolarization of cell membrane and can mediate both, excitatory or inhibitory actions *in vitro* (Flint et al., 1998; Kilb et al., 2002; Sava et al., 2014). However, as no evidence for synaptic glycine release has been observed in the immature neocortex, it has been proposed that taurine is the main endogenous ligand of this receptor (for review Kilb and Fukuda, 2017, but see Avila et al., 2013). In addition, activity-dependent reversal of the glycine transporter GlyT1, which is expressed at low levels in the immature cortex (Jursky and Nelson, 1996), can mediate a nonsynaptic glycine release pathway (Supplisson and Roux, 2002).

#### Other Neurotransmitter Systems

Information on the development of other neurotransmitter systems is less clear than for the classical neurotransmitters mentioned above. Cholinergic neurons and axons have been observed in the neocortical *anlage* at E16 (Schambra et al., 1989) and the acetylcholine (ACh) degrading enzyme ACh esterase can be detected at E15 in the VZ, CP and MZ of the neocortical *anlage* (Dori et al., 2005). The cholinergic agonist carbachol fails to induce any detectable response in cells in the VZ at E16 (LoTurco et al., 1995), but expression of functional nicotinic and muscarinic ACh receptors has been demonstrated in SP neurons of early postnatal rodents (Hanganu and Luhmann, 2004; Hanganu et al., 2009). Noradrenergic fibers have been also documented in the immature brain. At E18 they arrive in the neocortex and spread in the MZ, CP and SP (Levitt and Moore, 1979). Accordingly, α2 adrenoceptors have also been found in the VZ, CP and in migrating neurons of the embryonic brain (Winzer-Serhan and Leslie, 1999).

### Appearance of Activity Patterns during Cortical Development

One intriguing observation in developing neuronal networks, both *in vivo* and *in vitro*, is the appearance of spontaneous, mostly correlated neuronal activity (for review Spitzer et al., 2004; Khazipov et al., 2013; Luhmann et al., 2016; Luhmann and Khazipov, 2017). These spontaneous activity patterns reveal specific features during distinct developmental stages, in accordance with the establishment of neuronal connectivity (Egorov and Draguhn, 2013). The temporal sequence and properties of spontaneous neuronal activity in early postnatal animal models recapitulates the appearance of neural activity in human preterm babies (Tolonen et al., 2007). It is assumed that early postnatal stages in rodents are comparable to late fetal phases of human development (Clancy et al., 2007).

The earliest neocortical activity has been observed in the proliferative epithelium of the VZ and in the CP (Owens et al., 2000; Corlew et al., 2004). This early spontaneous activity is represented by Ca^2+^ transients, which differ in their properties and mechanisms between both regions. Ca^2+^ transients in the VZ are independent of electrical activity, rely on Ca^2+^ release from intracellular stores and depend on connexin hemichannels and purinoceptors (Owens et al., 2000; Weissman et al., 2004). They are not correlated between cells, with exception of occasionally synchronous pairs of cells, and are mostly probably generated in proliferating radial glial cells (Weissman et al., 2004). These uncorrelated Ca^2+^ transients in the proliferative VZ may at least partially resemble the Ca^2+^ signals that have been observed during mitosis in several cell types (Berridge, 1995; Santella, 1998). In contrast, Ca^2+^ transients in the CP are synchronized between neurons, are suppressed after inhibition of neuronal activity with the Na^+^ channel blocker tetrodotoxin (TTX), and rely on voltage-gated Ca^2+^ channels (Corlew et al., 2004). These findings indicate that electrical activity with subsequent Ca^2+^ influx is essential for the generation of CP Ca^2+^ transients.

**Figure 2:**
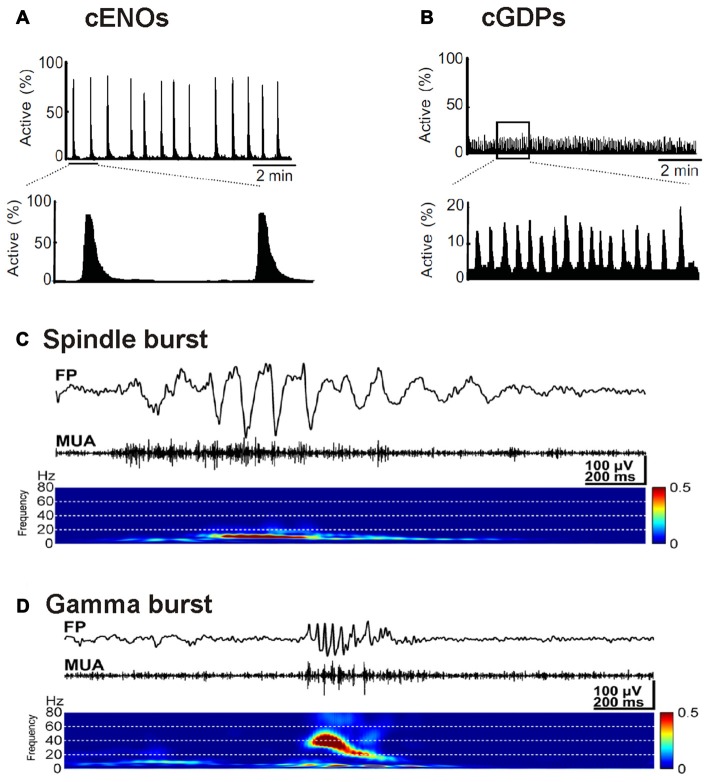
Examples of typical spontaneous activity patterns. **(A)** Spontaneous cortical early network oscillations (cENOs) in P3 rat neocortical neurons visualized by Ca^2+^ imaging. **(B)** In P6 rat neocortical slices, cortical giant depolarizing potentials (cGDPs) replace the cENOs. Note the higher frequency and the lower number of participating neurons in cGDPs (**A,B** modified from Allène et al., 2008). **(C)** Field potential recording of spindle bursts occurring spontaneously in the somatosensory cortex of a P1 rat *in vivo*. Below the FP trace the multi unit activity (MUA) identifies neuronal spikes and the wavelet analysis revealed that the spindle oscillations display a maximal frequency around 10 Hz. **(D)** Recording of a spontaneous gamma bursts in the somatosensory cortex of a P3 rat *in vivo* (**C,D** modified from Yang et al., 2009).

Large-scale electrical events emerge in the developing neocortex of rodents around birth (Garaschuk et al., 2000; Allène et al., 2008). These TTX-sensitive spontaneous events are termed cortical early network oscillations (cENOs), usually start in the posterior cerebral cortex, occur approximately every 2 min and propagate with ~2 mm/s over the whole cortex towards the anterior pole (Figure 2A). Recent experiments suggest that cENOs are triggered by a population of autonomously active layer 3 neurons (Namiki et al., 2013). Around postnatal day (P) 4–5, cENOs are gradually replaced by cortical giant depolarizing potentials (cGDPs). These cGDPs occur at higher rates (~8 min^−1^) and show substantially faster kinetics (Figure 2B; Allène et al., 2008). In the visual cortex the majority of spontaneous events is classified as slow activity transients (SATs), long lasting (~10 s) events characterized by a summation of rapid oscillatory bursts (15–30 Hz), occurring after P10 and before eye opening (Colonnese and Khazipov, 2010). SATs disappear after enucleation (Colonnese and Khazipov, 2010), indicating that retinal waves drive this spontaneous activity pattern. Retinal waves are spatially highly correlated spontaneous activity patterns that depend on cholinergic and in later phases also on glutamatergic neurotransmission (Zhou and Zhao, 2000).

In addition to these patterns that synchronize large neocortical areas, a variety of activity transients that mediate local synchronization has been described (for review Khazipov et al., 2013; Luhmann and Khazipov, 2017). In visual and somatosensory areas of the early postnatal (P0–3) neocortex, local and short network oscillations in a frequency range of 10–20 Hz have been recorded (Khazipov et al., 2004; Hanganu et al., 2006; Minlebaev et al., 2007; Yang et al., 2009, 2013). These short events (0.5–3 s) occur spontaneously every ~10 s and have been termed spindle bursts due to their typical appearance in electrophysiological recordings (Figure 2C; for review Yang et al., 2016). *In vitro* (Dupont et al., 2006; Hanganu et al., 2009) as well as *in vivo* data (Yang et al., 2009; Tolner et al., 2012) suggest that SP neurons are involved in the generation or amplification of spindle bursts (for review Luhmann et al., 2009; Kanold and Luhmann, 2010). A substantial part of the spindle oscillations reflects activity in the immature sensory periphery (for review Yang et al., 2016). In neonatal rat primary somatosensory cortex, spatially confined spindle bursts are triggered by spontaneous muscle twitches (Khazipov et al., 2004), which have been observed during intrauterine development in several mammalian species including humans (Khazipov et al., 2004; Tiriac et al., 2014). In somatosensory cortex of premature human babies delta brushes, which have been proposed to be the EEG correlates of spindle bursts, are induced by slight mechanical stimulation, indicating that already at prenatal stages sensory inputs affect cortical activity (Milh et al., 2007). A similar peripheral origin for activity has also been observed for the visual cortex, where spindle bursts and SATs depend on retinal waves (Hanganu et al., 2006; Colonnese and Khazipov, 2010). In the auditory system spontaneous network bursts are recorded in the auditory cortex (Kotak et al., 2007), while also in the periphery spontaneous Ca^2+^ action potentials occur in fetal inner hair cells of the cochlea (Marcotti et al., 2003). This early spontaneous cochlear activity is synchronized via ATP-dependent signaling and triggers theta-like bursting activity in auditory nerve fibers (Tritsch et al., 2007), which initiate spontaneous burst activity in the auditory brainstem before hearing onset (Tritsch et al., 2010). Taken together, these observations indicate that a substantial proportion of spontaneous, spindle burst activity observed in the developing sensory neocortex is caused by events in the sensory periphery or intermediate relay stations (for review Luhmann et al., 2016).

Around P3, spindle bursts are complemented by spontaneous gamma oscillations with a higher frequency of 30–40 Hz (Figure 2D). They typically have a duration of 100–300 ms and are restricted to local functional columns (Yang et al., 2009; Minlebaev et al., 2011; Khazipov et al., 2013). Interestingly, they are only marginally affected by inhibitory synaptic transmission, indicating that they are functionally distinct from gamma oscillation observed in mature neocortex (Khazipov et al., 2013). At least in the somatosensory system of newborn rodents, gamma oscillations can be recorded in the thalamus and synchronize a single thalamic barreloid with the corresponding neocortical barrel (Minlebaev et al., 2011; Yang et al., 2013), indicating that thalamocortical interactions contribute to the generation of gamma oscillations already in the immature neocortex. Recent experiments demonstrated that neurons in supragranular layers of the developing neocortex are particular capable to generate high frequency oscillatory events (Bitzenhofer et al., 2017).

However, inhibition of peripheral sensors and/or peripheral nerves abolishes only a fraction of spindle bursts and gamma oscillations in the somatosensory cortex of early postnatal mice (Yang et al., 2013; An et al., 2014), suggesting that a substantial proportion of spontaneous activity is generated in subcortical nuclei or within the developing neocortex itself. Such central spontaneous activity transients can directly interfere with activity in adjacent cortical areas independent of thalamic connections (Zagha et al., 2013).

### Role of Neurotransmitter Systems in Spontaneous Activity

In particular during early developmental stages spontaneous events seem to be influenced by electrical synapses (Blankenship and Feller, 2010). *In vitro* correlates of spindle bursts at early postnatal phases are independent of GABA and glutamate, but require gap junctional coupling (Dupont et al., 2006), suggesting that electrical synapses are essential for the generation of oscillatory activity during these stages. Interestingly, this activity undergoes a fast developmental switch at ~P4 when this activity depends mainly on AMPA receptors (Dupont et al., 2006). This observation is consistent with the observation that at comparable developmental stages glutamatergic inputs and local GABAergic interneurons mediate spindle burst firing also *in vivo* (Bitzenhofer et al., 2015).

The cENOs depend on both AMPA and NMDA receptors, demonstrating the glutamatergic nature of these events (Garaschuk et al., 2000; Allène et al., 2008). In contrast, cGDPs are only partially affected by AMPA/NMDA receptor antagonists, but depend on GABA_A_ receptor-mediated transmission (Allène et al., 2008). The peripherally generated portion of spontaneous spindle and gamma bursts in the cortex depends on thalamocortical synaptic connections, either directly or via SP neurons, and thus on glutamatergic synapses characteristic of these connections (Zhao et al., 2009).

Activation of muscarinic ACh receptors induces *in vitro* correlates of spindle-like spontaneous activity (Dupont et al., 2006; Hanganu et al., 2009). In line with this, electrical stimulation of the cholinergic basal forebrain nuclei induces spindle busts and inhibition of ACh degradation by the ACh esterase inhibitor physostigmine enhances the occurrence of spontaneous spindle bursts *in vivo* (Hanganu et al., 2007; Janiesch et al., 2011). In contrast, inhibition of muscarinic ACh receptors reduces the occurrence of spindle busts in the visual cortex (Hanganu et al., 2007). These results imply that the cholinergic system may be relevant for the generation of spontaneous activity in neocortical circuits. Another neurotransmitter system that is important for the generation of spontaneous activity is the purinergic system. In the VZ (Weissman et al., 2004) and the olfactory system (Tritsch et al., 2007) a critical role of purinoceptors and/or ATP as relevant neuromodulator has been shown, indicating that this neurotransmitter system is highly relevant for the generation of ontogenetically early activity.

## Impact of Neuronal Activity on Neurodevelopmental Events

There is increasing experimental evidence that the above-mentioned early patterns of spontaneous neuronal activity are not an epiphenomenon reflecting the gradual maturation of membrane excitability, neuronal connectivity or synaptic functions, but rather that these spontaneous activity patterns play an important role in various neurodevelopmental processes. Even in clinical setting it has been demonstrated that the properties of spontaneous activity recorded with EEG in preterm infants provide prognostic value of brain activity for further development (Iyer et al., 2015).

### Neurogenesis

A series of influential studies demonstrated that neurogenesis is directly governed by neurotransmitters. In the VZ glutamate, acting on AMPA/kainate receptors, and GABA, acting on GABA_A_ receptors, stimulates neurogenesis, while inhibition of AMPA/kainate or GABA_A_ receptors promotes neurogenesis (LoTurco et al., 1995). In the SVZ, which is another proliferative part of the developmental neocortex, the effect of glutamate and GABA on neurogenesis is opposite (Haydar et al., 2000). These results indicate that both neurotransmitter systems are directly involved in the regulation of neurogenesis. In addition, glycine receptors and muscarinic ACh receptors modulate the rate of neurogenesis in the neocortical *anlage* (Ma et al., 2000; Avila et al., 2014). While these previous observations only indicate an influence of interstitial neurotransmitter on neurogenesis, without identifying the source of these neurotransmitters and the mechanisms of their action, more recent results indicate that neuronal activity is directly involved in the regulation of neurogenesis. Ca^2+^ transients occur during mitosis in several cell types and probably directly influence proliferation (Berridge, 1995; Santella, 1998), suggesting that neuronal activity and/or non-synaptic GABA/glutamate release mediate their effect on neurogenesis by interference with these Ca^2+^ transients. In the VZ of mice, the frequency and spatial properties of Ca^2+^ waves are directly correlated to the amount of neurogenesis (Weissman et al., 2004), suggesting that such activity transients may modulate the rate of neurogenesis. A more causal evidence comes from experiments in which the suppression of Ca^2+^ waves by antagonists of purinoceptors reduces proliferation in the VZ (Weissman et al., 2004), suggesting that spontaneous Ca^2+^ waves can be directly involved in the regulation of neurogenesis. In the visual cortex, pharmacological inhibition of retinal waves increases neurogenesis (Bonetti and Surace, 2010), demonstrating for the first time that also peripherally generated activity is correlated to neurogenesis. An additional hint that neuronal activity might enhance neurogenesis comes from clinical studies in preterm babies, where increased activity in EEG recordings during early developmental stages is correlated with faster growth of brain structures during subsequent months until term age (Benders et al., 2015). However, in this study it could not be excluded that: (i) neuronal activity affects further developmental processes influencing the size of brain structures (e.g., by acting on apoptosis); or (ii) that underlying pathophysiological mechanism have independent effects on both immature activity and further brain development, thereby creating spurious relations. Further experiments are required to evaluate to which extent the effect of the diverse neurotransmitters on neurogenesis is modulated by an interference with spontaneous Ca^2+^ transients in the VZ.

### Determination of Neuronal Identity

Neuronal activity directly influences the expression pattern of various factors and thereby influences the phenotype of neurons (Flavell and Greenberg, 2008). Spontaneous oscillatory activity modulates e.g., the expression of transcription factors (Hanson and Landmesser, 2004), which can subsequently change the phenotype of neurons. A series of studies provided evidence that the neurotransmitter phenotype of neurons is rather plastic and can be modified by electrical activity (for review Spitzer, 2017). In spinal neurons from *Xenopus*, the frequency of spontaneous Ca^2+^ transients is positively correlated with the number of GABAergic neurons, while inhibition of Ca^2+^ transients promotes the generation of excitatory neurons (Gu and Spitzer, 1995; Borodinsky et al., 2004). However, to our knowledge, a homeostatic change in neurotransmitter phenotype has not been demonstrated in the developing neocortex (for review Spitzer, 2017), despite the fact that major properties required for “neurotransmitter plasticity” are present in the mammalian CNS (Demarque and Spitzer, 2012). Other experiments demonstrated that even the expression of basic morphogenic factors like sonic hedgehog (Belgacem and Borodinsky, 2015) is directly regulated by electrical activity. Further studies regarding this interesting issue are required to uncover, whether spontaneous electrical activity is an important determinant of global neurodevelopmental processes, e.g., for area specification (for review Yamamoto and López-Bendito, 2012).

### Neuronal Migration

Neuronal migration critically depends on patterned activity, as has been demonstrated in the seminal experiments by Komuro and Rakic (1996), who observed *in vitro* that repetitive Ca^2+^ transients in neurons are directly linked to forward migration. Impairment of these repetitive Ca^2+^ transients by Ca^2+^ channel agonists (Komuro and Rakic, 1996), by inhibition of neuronal activity (Heck et al., 2007) or by the expression of hyperpolarizing K^+^ channels (De Marco García et al., 2011) attenuates neuronal migration. In addition, a variety of studies demonstrated that various neurotransmitters influence neuronal migration (for review Luhmann et al., 2015).

In a series of experiments Toby Behar and Jeffery Walker uncovered the essential role of GABA for neuronal migration. In summary, these *in vitro* experiments with dissociated cell and organotypic cultures demonstrated that GABA acts as chemokine/chemoattractant and that inhibition of GABA_A_ receptors enhances the number of postmitotic neurons reaching the CP, while the unspecific GABA_A/C_ antagonist picrotoxin induces a transient reduction in postmitotic neurons in the CP (Behar et al., 1996, 2000). These results led to the hypothesis that GABA_A_ receptors provide a “stop signal” for migrating neurons, while GABA_C_ receptors are required as a pro-migratory stimulus. Indeed, it could later be verified that a specific inhibition of GABA_C_ receptors impedes radial neuronal migration (Denter et al., 2010). In line with these *in vitro* results, focal inhibition of GABA_A_ receptors *in vivo*, using small elvax implants, induces ectopic neurons in superficial neocortical layers that resemble a hypermigratory phenotype (Heck et al., 2007). Intriguingly, a similar phenotype has been also observed after *in vivo* application of elvax implants containing the GABA_A_ receptor agonist muscimol. This observation is due to the fact that continuous presence of muscimol leads to a massive desensitization of GABA_A_ receptors and/or impairs the repetitive Ca^2+^ transients required for adequate migration (Heck et al., 2007).

*In vitro* experiments demonstrated that activation of glycine receptors also impairs radial migration, while inhibition of glycine receptors is without effect, indicating that the glycinergic system has the potential to influence migration, but is not directly involved in the regulation of radial migration in the early postnatal neocortex (Nimmervoll et al., 2011). Genetic deletion of the glycine receptor subunit α_2_, which is a major α subunit expressed in perinatal cerebral cortex (Malosio et al., 1991), reduces the number of interneurons migrating from the SVZ into the cortex at E15 (Avila et al., 2013). However, it has been demonstrated that activation of glycine receptors enhances GABAergic network activity in the immature neocortex (Sava et al., 2014). Thus it is still unresolved whether activation of glycine receptors is directly linked to neuronal migration or whether glycinergic effects on network activity are the crucial parameter influencing these migratory events.

The glutamatergic system is also directly involved in the modulation of neuronal migration. Both, *in vitro* (Behar et al., 1999) and *in vivo* (Reiprich et al., 2005) experiments demonstrated that inhibition of NMDA receptors disturbs neuronal migration. Experiments performed in organotypic slice cultures of the cerebral cortex revealed that both activation (Kihara et al., 2002) and inhibition (Hirai et al., 1999) of NMDA receptors between E16 and E18 slow down cell migration, indicating that moderate activation of NMDA receptors by endogenous glutamate may be required for adequate responses. However, NMDA receptor-mediated effects on cell migration are not changed in slices obtained from munc18-1 knock-out animals, in which synaptic neurotransmitter release has been deleted (Manent et al., 2005), challenging the physiological role of neurotransmitters as regulators of migration. As electrical synapses and non-vesicular release processes play an important role during neuronal development, and since a variety of neurodevelopmental events are directly affected by neurotransmitters or lack of neuronal activity, it can be assumed that non-synaptically mediated activity is sufficient for early neurodevelopmental events (Manent et al., 2005).

Other neurotransmitter systems are also affecting neuronal migration. Activation of D1 dopamine receptors stimulates, while D2 receptor activation impedes tangential migration of GABAergic interneurons (Crandall et al., 2007). The observation that the suppression of a radial glial cell specific splice variant of the ACh esterase attenuates radial migration (Dori et al., 2005) indicates that the cholinergic system also contributes to the regulation of neuronal migration.

Overall, these results clearly demonstrate the essential role of various neurotransmitters as regulators of neuronal migration, but how much of the mostly pharmacologically induced effects are mediated by direct interactions with migrating neurons, and to which extent secondary influences on neuronal activity contribute to these effects remain to be investigated in more detail. Recent studies revealed a direct influence of neuronal activity on neuronal migration. Pharmacological inhibition of retinal waves disturbs the adequate layer formation in the visual cortex (Bonetti and Surace, 2010), suggesting that peripheral activity patterns directly control the structural maturation of the neocortex.

### Neuronal Apoptosis

**Figure 3:**
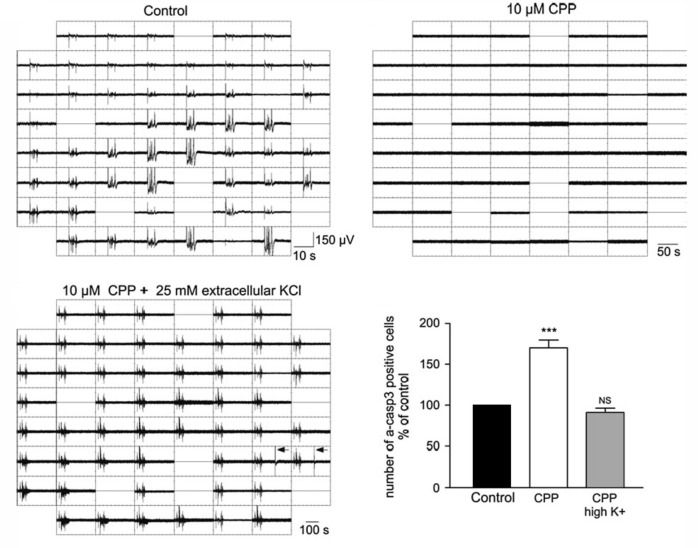
Influence of neuronal activity on apoptosis. Electrophysiological recordings and determination of apoptotic cells by activated Caspase3 immunohistochemistry in dissociated cortical cultures reveal that the NMDA receptor antagonist CPP blocks neuronal activity and enhanced apoptosis (***p < 0.001, Kruskal-Wallis test). This effect of CPP on apoptosis can be rescued in the presence of high K^+^ concentration, which restores the electrical activity (modified from Heck et al., 2008).

Neuronal apoptosis, i.e., the programmed cell death of neurons, is a fundamental process in the developing brain, which determines the final size and organization of mature neuronal assemblies (for review Kuan et al., 2000; Blanquie et al., 2017a). A number of experiments demonstrated that apoptosis is modified by electrical activity. Silencing of cortical networks with TTX increases the number of apoptotic neurons *in vitro* (Ruijter et al., 1991; Heck et al., 2008; Schonfeld-Dado and Segal, 2009; Golbs et al., 2011), as well as *in vivo* (Murase et al., 2011). On the other hand, the number of apoptotic neurons *in vitro* decreases when spontaneous activity is enhanced by elevated extracellular K^+^ concentrations (Ghosh et al., 1994), by inhibition of GABA_A_ receptors (Léveillé et al., 2010), or by low NMDA doses (Soriano et al., 2006). In line with the last observation, inhibition of NMDA receptors triggers apoptosis *in vivo* (Ikonomidou et al., 1999). A direct link between electrical activity and neuronal survival has been demonstrated by simultaneous recordings of electrical activity and monitoring cell death in dissociated neuronal cultures. In neocortical cultures, high-frequency neuronal activity attenuates caspase-3 dependent apoptosis (Figure 3; Heck et al., 2008). The dependence of apoptotic rates on electrical activity could be quantified in experiments with hippocampal neurons, which revealed that inactive neurons are ten times more likely to undergo apoptosis than neurons contributing to spontaneous, synchronous network activity (Murase et al., 2011). Since it has been reported that both, immature activity patterns (Yang et al., 2009; Minlebaev et al., 2011) and the distribution of apoptosis (Ikonomidou et al., 1999; Stankovski et al., 2007) show an uneven distribution across regions and layers of the developing cortex, the amount and pattern of spontaneous neuronal activity might directly influence the structural development of the neocortex by regulating apoptosis rates. This hypothesis has recently been addressed by a study showing that spontaneous and periphery-driven activity patterns refine the final number of cortical neurons in a region-dependent manner and thus can directly influence the structural and functional development of the neocortex (Blanquie et al., 2017c).

For neocortical dissociated cultures it has been shown that the activity-dependent release of brain-derived neurotrophic factor (BDNF) and activation of phosphatidylinositol 3-kinase are key downstream elements in the activity-dependent apoptosis pathway, which promotes survival via the AKT pathway (Wagner-Golbs and Luhmann, 2012). However, depending on the pattern of electrical activity or the site of calcium entry other pathways are probably also involved in the activity-dependent regulation of neuronal apoptosis (for review Hardingham and Bading, 2010). Suppression of neuronal activity with ethanol or antiepileptic drugs has also been shown to induce apoptosis during early development *in vivo* (Ikonomidou et al., 2000; Bittigau et al., 2002; Ikonomidou and Turski, 2010; Lebedeva et al., 2017), providing further support that a modification of physiological activity patterns during early development affects neuronal apoptosis. Finally, impairment of presynaptic neurotransmitter secretion in Munc18-1 deleted animals results in a drastic apoptotic response in subcortical areas around the developmental stage when initial synaptic responses should occur (Verhage et al., 2000). Unfortunately, no indication for neocortical apoptosis could be observed in these studies, as these animals die perinatally before the wave of neocortical apoptosis occurs. In this respect it should be considered, that the effect of neuronal activity and/or neurotransmitter on neuronal apoptosis is specific for particular neuronal populations. Recently it has been demonstrated that the transient Cajal-Retzius neurons could be rescued from apoptosis by inhibition of depolarizing GABAergic responses, while the rate of apoptosis in the remaining cortical neurons was unaltered under this condition (Blanquie et al., 2017b). Interestingly, the noradrenergic system also has opposite effects on the rate of apoptosis in Cajal-Retzius cells (Naqui et al., 1999) as compared to other neocortical neurons (Popovik and Haynes, 2000).

The observation that neuronal activity regulates the rate of apoptosis raises the intriguing question whether only the level of neuronal activity or the specific pattern of activity may mediate the pro-survival effect. Direct evidence that the pattern of activity regulates neuronal apoptosis is provided from experiments using organotypic slice cultures (Heck et al., 2008). Inhibition of gap junctions desynchronizes the typical spontaneous network activity, but increases the overall frequency of action potentials. From the observation that under this condition the rate of apoptosis is increased (Heck et al., 2008), it was concluded that synchronicity of discharges is a more important factor for the suppression of neuronal apoptosis than the number of discharges. Subsequent experiments revealed that a moderate increase in extracellular K^+^ concentration, which slightly enhances the frequency of correlated bursts, or the induction of highly synchronized activity after inhibition of GABA_A_ receptors also reduces the number of apoptotic neurons (Golbs et al., 2011). However, Golbs et al. (2011) also revealed that a further increase in the K^+^ concentration causes a desynchonization of neuronal activity with the appearance of high frequency tonic firing, which still provide a strong antiapoptotic signal, suggesting that other factors beyond the appearance of synchronous bursts also contribute to activity-dependent neuronal survival. Therefore, further experiments elucidating the “neuronal code” that provides antiapoptotic effects are required to fully understand the relation between neuronal activity and survival.

### Establishment of Mature Neuronal Connectivity

It is often assumed, that the major organization of neuronal connectivity is genetically encoded and controlled by the spatio-temporal pattern of transcription factors as well as by intercellular communication via cell-cell interactions or constitutive secretion of growth factors (Chao et al., 2009). The most stringent evidence for activity-independent formation of connectivity comes from SNAP-25 deficient mice, in which vesicular neurotransmitter release and thus network activity is completely abolished, but the prenatal formation of thalamocortical connections is maintained (Molnár et al., 2002).

A variety of studies demonstrated that spontaneous activity influences growth and differentiation of neuronal dendrites and axonal projections and is essential for the formation of mature functional neuronal circuits (for review Chen and Ghosh, 2005; Zheng and Poo, 2007; Yamamoto and López-Bendito, 2012). Many of these studies have been performed on developing visual cortex, in which the organization of ocular dominance columns serves as a model for the establishment of adequate connectivity (Ackman and Crair, 2014). Already the early studies of Hubel and Wiesel demonstrated that correlated visual activity during critical periods represents a prerequisite for adequate connectivity (Hubel and Wiesel, 1970). Inhibition of early spontaneous retinal activity, which provides correlated inputs from the sensory periphery before eye opening, causes a persistent disorganization of ocular dominance columns and a pronounced increase in receptive field size of neurons in primary visual cortex (Huberman et al., 2006). Another causal evidence for the role of spontaneous activity is provided by *in vivo* experiments, which used optogenetic manipulation of retinal activity to demonstrate that patterned retinal activity, which is asynchronous and has a delay of >100 ms between both eyes, is required for the development of eye segregation (Zhang et al., 2012). Interestingly, spontaneous retinal activity directly influences the expression of several components of the ephrin system, which is essential for the development of adequate neuronal connections in the visual system (Nicol et al., 2007). This result provides a mechanism by which electrical activity can influence the outgrowth of axons. In addition this suggests that molecular cues controlling neocortical connectivity, which are typically considered as activity-independent factors in early development, may at least partially depend on neuronal activity.

**Figure 4:**
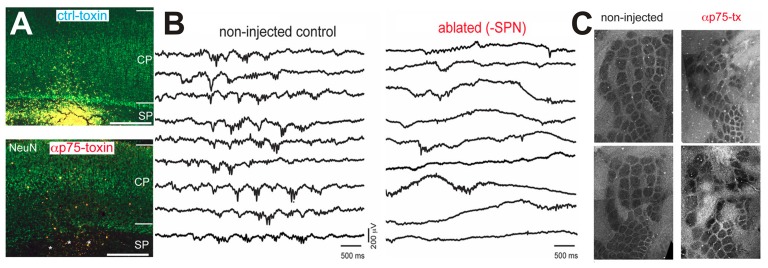
Impairment of neuronal activity affects structural formation of the neocortex. **(A)** The injection of the neurotoxin αp75 selectively ablates SP neurons. **(B)** Ablation of SP neurons suppresses the occurrence of spontaneous spindle busts in the somatosensory cortex. **(C)** Cytochrome-oxidase stained tangential slices of P10 mice indicate that the barrel formation is severely impaired after ablation of SP neurons (modified from Tolner et al., 2012).

In the immature somatosensory cortex a stringent role of spontaneous activity in the formation of functional neuronal circuits has been found. Using a comparable experimental approach as Hubel and Wiesel, trimming of mystacial whiskers during a critical period impaired the formation of “whisker barrels” in layer 4 (Woolsey and Wann, 1976), which are the neocortical representations of single whiskers in the primary somatosensory cortex. In the barrel cortex of early postnatal rats spontaneous activity also appears before the onset of active whisking (Yang et al., 2009) and a considerable portion of this correlated spontaneous activity is independent of activity in the sensory periphery (Yang et al., 2013). Intriguingly, in early postnatal rat the spatial domains of the spontaneous oscillations resemble the characteristic columnar organization of neocortical whisker representations several days before the anatomical correlate of these whisker representations become obvious (Yang et al., 2013; Luhmann, 2017). Attenuation of spontaneous spindle bursts activity by ablation of the SP using p75-immunotoxin severely impairs the columnar organization of the rodent barrel cortex (Figure 4; Tolner et al., 2012), providing causal evidence that spontaneous and locally synchronized neuronal activity is essential for the functional maturation of neocortical architecture in the barrel cortex.

In the auditory cortex, peripheral activity also shapes the maturation of neocortical representation (Chen and Yuan, 2015). Repetitive presentation of pure tones during the critical period after onset of hearing massively shifts the tonotopic map, while application of white noise disrupts the formation of tonotopy (de Villers-Sidani et al., 2007; Zhou et al., 2008). In the developing auditory system, it has been also shown that correlated, patterned spontaneous activity before the onset of hearing is required for the formation of the mature, functional connectivity at least in brainstem nuclei (Clause et al., 2014).

In summary, these results suggest that spontaneous neuronal activity present before the appearance of adequate sensory stimulation is required for the normal development of the cortical architecture in primary sensory areas. Neuronal activity plays also an important role in the subsequent functional maturation of initially formed synaptic networks. First, spontaneous synchronous network activity is required to activate silent synapses by incorporating AMPA receptors into the postsynaptic membrane (Durand and Konnerth, 1996; Voigt et al., 2005), thereby shaping the functional connectivity within the existing structural network. Second, neuronal activity plays an essential role in the pruning of redundant neuronal connections (e.g., Hata and Stryker, 1994; for review Wong and Ghosh, 2002).

### Axon Myelination

The myelination of axons, required for fast spike propagation, is also directly controlled by neuronal activity. Both, sensory evoked responses as well as spontaneous neuronal activity, regulate axon myelination (Demerens et al., 1996; Barrera et al., 2013). Neuronal activity causes activation of adenosine receptors on oligodendrocyte precursor cells, which subsequently enhances the proliferation and differentiation of these cells (Stevens et al., 2002). In addition, the activity-dependent release of glutamate from active axons directly induces myelin formation in differentiated oligodendrocytes (Wake et al., 2015), which is mediated via activation of NMDA receptors and a subsequent Fyn-kinase dependent release of translational repression (White and Krämer-Albers, 2014). However, to our knowledge it has not been investigated whether single action potentials or patterned activity is required for proper activation of oligodendrocytes and myelination.

### Non-neuronal Components of the Central Nervous System

Non-neuronal components of the central nervous system are also affected by electrical activity. As mentioned above, neuronal activity initiates the differentiation of oligodendrocytes via the axonal release of adenosine (Stevens et al., 2002). Immature astrocytes under *in vitro* conditions also demonstrate massive alterations in gene expression profile if the activity levels on co-cultured neurons are altered (Hasel et al., 2017). Furthermore, it has been recently shown that peripheral-driven neuronal activity also regulates vessel development and patterning in the developing rodent brain (Whiteus et al., 2013; Lacoste et al., 2014). In summary, these observations strongly indicate that neuronal activity can shape the structural development of the neocortex also by influencing non-neuronal components.

## Pathophysiological Implications of Disturbed Perinatal Neuronal Activity

The influence of neuronal activity patterns on various developmental events is also supported by clinical studies. We concentrate our review: (i) on studies demonstrating that conditions leading to disturb neuronal activity cause neurodevelopmental diseases; and (ii) on diseases in which putative alterations in immature neuronal activity have been associated with their clinical manifestation.

### Substances That Interfere with Early Neuronal Activity

One intriguing observation supporting the role of neuronal activity in the structural and functional development of the neocortex is that substances interfering with early neuronal activity often cause disturbances in neocortical development. Recently, a pilot study performed in preterm babies (26–33 postconceptional weeks) revealed that several drugs (e.g., fentanyl, phenobarbital, theophylline) that are routinely used in neonatal intensive care units alter the properties of spontaneous brain activity (Malk et al., 2014), suggesting that the impact of spontaneous activity patterns in early human development should also be considered to evaluate possible adverse effect of such substances.

The majority of anesthetics suppress neuronal activity by acting either as NMDA antagonists, GABA mimetics or gap junction blockers. Several animal studies demonstrated that such anesthetics can induce severe neurodegeneration in the immature brain. Exposure to isoflurane at a clinical relevant concentration (1.5 vol. % for 6 h) leads to a widespread neurodegeneration in the cortex of immature rats (Jevtovic-Todorovic et al., 2003). Both, the NMDA antagonist ketamine (20–50 mg/kg) and the GABAergic agonist midazolam (9 mg/kg) induced a neuroapoptotic response after 5 h treatment in neonatal mice, which was enhanced upon combination of both substances (Young et al., 2005). Recently it could be shown that the combined application of 40 mg/kg ketamine and 9 mg/kg midazolam indeed reduced spontaneous activity levels by ca. 90%, while sensory evoked neuronal activity was less effected, suggesting that the attenuation of spontaneous activity may be more relevant for the neuroapoptotic responses occurring under this conditions (Lebedeva et al., 2016). While N_2_O (50%–75% for 6 h) does not induce obvious neurodegeneration in rats (Jevtovic-Todorovic et al., 2003), the combination of N_2_O (70 vol. %) and isoflurane (0.75 vol. % for 6 h) results in a significant increase in the number of apoptotic neurons in rats (Zhou et al., 2011). Finally, 5 h application of isoflurane (1–1.5 vol. %) also induces apoptosis in fetal Rhesus macaque brains *in vivo* (Creeley et al., 2014). In cultures from human neural stem cells (hNSCs) it was shown that ketamine (100 μM) induced neuronal apoptosis, but only after a prolonged application of 24 h (Bai et al., 2013), while isoflurane (5%) application for 4 h does not affect neuronal survival in these human neuronal cultures (Sohn et al., 2017). Both results may suggest that human neurons are less prone to anesthetic-induced neuronal apoptosis. In accordance with these *in vitro* data, a recent multicenter study failed to observe a systematic adverse outcome in 2 year old infants that underwent a general servoflurane (median 54 min) anesthesia in the first postnatal weeks (Davidson et al., 2016). This observation is in line with the results of other clinical studies that reported no systematic cognitive outcomes of anesthesia during early childhood, which however typically include anesthesia in slightly older children (<3–4 years; for review O’Leary and Warner, 2017). In addition, it should be emphasized that at least the adverse effects of ketamine may not directly be related to reduced neuronal activity, since ketamine directly interacts with the mitochondrial potential (Ito et al., 2015).

Several anticonvulsants that interact with GABA_A_ and glutamate receptors and therefore suppress neuronal activity are also associated with an exacerbated apoptotic response in early postnatal rodents (Bittigau et al., 2002; Ikonomidou and Turski, 2010). The anticonvulsant drugs vigabatrin (200 mg/kg/day), valproate (100 mg/kg/day) and lamotrigine (20 mg/kg/day) can cause severe cortical malformations upon fetal exposure (Manent et al., 2007, 2008) and fetal valproate exposure (600 mg/kg at E12.5) disturbs the formation of tonotopic maps (Dubiel and Kulesza, 2015), suggesting that these substances can in animal experiments impair neuronal migration and functional differentiation. Also in cultures of hNSCs chronic application of the anticonvulsant substances phentobarbital (10 μM), valproate (10 μM), lamotrigine (1 μM) or carmazepine (1 μM) reduces cell viability (Cao et al., 2015). In humans, fetal exposure to these drugs is known to trigger long-term structural, behavioral and cognitive defects including modification of the brain mass, learning and memory impairments, anxiety, social interaction and sensorimotor integration disabilities (for review Turski and Ikonomidou, 2012). Since anticonvulsants attenuated spontaneous brain activity in preterm babies (Malk et al., 2014), we suggest that, in addition to other mechanisms (for review Schachtner, 2017), reduced neuronal activity should be considered as putative mechanism for the teratogenic effects of anticonvulsants.

One major clinical implication emerging from these advances in the understanding of the impact of neuronal activity on the structural and functional development of the neocortex is that the use of pharmacological therapies that interfere with neuronal activity during early developmental stages (i.e., during pregnancy or during early postnatal phases) must be carefully evaluated.

#### Fetal Alcohol Syndrome

The clinically most relevant case for pharmacologically induced neurodevelopmental disorder is still the fetal alcohol spectrum disorder (FASD), caused by ethanol consumption during pregnancy (for review Olney, 2014). Excessive ethanol consumption during a specific developmental stage which coincides with synaptogenesis in the cortex (prenatally in humans and early postnatally in rodents) induces apoptotic neurodegeneration that leads to gross structural brain pathology and a variety of long-term behavioral, cognitive or neuropsychiatric disorders (Olney et al., 2002). Although originally ethanol has been suggested to affect predominantly neurons, ethanol-induced apoptosis of glial cells has been also reported (for review Guizzetti et al., 2014). Long term exposure to ethanol (20 mM, 1 week) increases apoptosis in undifferentiated hNSCs and suppresses astrocyte differentiation, while neuronal differentiation is unaffected (Nash et al., 2012). Thus a loss of astrocytes may partially underlie ethanol neurotoxicity. These results can be explained by ethanol effects on gene expression in hNSCs (Khalid et al., 2014). The devastating effects of ethanol on developing brains has been attributed to a variety of mechanism from genetic and epigenetic alteration to metabolic and nutritional disturbances (for review Muralidharan et al., 2013), however, direct effects of ethanol on neuronal activity may contribute to the FASD (for review Lotfullina and Khazipov, 2017).

Ethanol inhibits NMDA receptor-mediated signals and enhances GABA_A_ receptor-mediated responses (Lovinger et al., 1989; Ikonomidou et al., 2000; Glykys et al., 2007), therefore it should suppress neuronal network activity in the brain. However, *in vitro* results obtained in acute hippocampal slices of P3–7 rats demonstrated that ethanol significantly increases the frequency of giant depolarizing potentials (GDPs; Galindo et al., 2005), supporting the hypothesis that GABA_A_ receptors mediate excitatory actions (Ben-Ari et al., 2012). However, *in vivo* GABA_A_ receptors provide a dominant inhibitory action already in early postnatal rodent cortex (Kirmse et al., 2015). In line with this, recent *in vivo* recordings in neonatal rats demonstrated that at P4–7 alcohol almost completely suppresses gamma oscillations and spindle bursts as well as sensory-evoked responses, whereas its action at P14–17 was much weaker (Lebedeva et al., 2017). In parallel to these electrophysiological observations it has been documented in the same study that the strongest apoptotic effect of ethanol occurs between P4 and P7, while already at P14–17 ethanol fails to increase apoptotic cell death (Lebedeva et al., 2017), suggesting that *in vivo* an alcohol-induced reduction of neuronal activity appears to contribute to its neurodegenerative effect (for review Lotfullina and Khazipov, 2017). In addition, it has been shown that ethanol also directly impairs the frequency of Ca^2+^ transients in migrating mouse neurons and the rate of neuronal migration (Kumada et al., 2006). This attenuation of neuronal migration may also contribute to the deleterious effects of ethanol consumption during pregnancy.

### Neonatal Encephalopathy

The most common cause of neonatal encephalopathy is perinatal asphyxia, a relevant complication occurring in about 2% of human births. Consequences are several brain dysfunctions like cerebral palsy, sensory and cognitive impairments, epilepsy or autism spectrum disorders (ASD; Vannucci, 2000). Other causes, like sepsis, meningitis or metabolic disturbances have been also discussed as possible causes underlying neonatal encephalopathy. While it is obvious that hypoxic-ischemic periods and/or severe infections impair the development of the cortex by many mechanisms, e.g., by excitotoxicity or apoptosis (Vannucci, 2000), recent experiments suggest that direct and persistent effects of neonatal encephalopathy on neuronal activity may be also considered as potential factors leading to neonatal encephalopathy. A variety of studies demonstrated that hypoxic and/or ischemic conditions in the immature brain induce acute and also long-lasting changes in spontaneous activity in humans (Roberts et al., 2014) and in rodents, both *in vitro* and *in vivo* (Brockmann et al., 2013; Ranasinghe et al., 2015; Mordel et al., 2016).

*In vitro* experiments revealed that immature rodent cortical neurons are more resistant against hypoxic-ischemic conditions (Luhmann and Kral, 1997), whereas SP neurons might be more vulnerable to hypoxia (McQuillen and Ferriero, 2005; but see Albrecht et al., 2005; Okusa et al., 2014). Due to their important role for spontaneous activity and cortical rewiring (Dupont et al., 2006; Tolner et al., 2012), loss of SP neurons can particularly contribute to the long lasting impairment of spontaneous and neuronal activity. In addition, experimentally induced inflammation induces in rodents a rapid modification in the properties of spontaneous activity *in vitro* and *in vivo* (Nimmervoll et al., 2013), thus indicating that conditions like bacterial or viral infections may also have an impact on cortical development by modifying neuronal activity. While it is obvious that both hypoxic-ischemic insults as well as inflammation directly impair neuronal survival by various mechanisms (Millar et al., 2017), we suggest that persistent effects of such insults on spontaneous neuronal activity patterns may be an additional factor contributing to acute and chronic neuronal dysfunction.

### Epilepsy

Epilepsy is one of the most common neurological disorders. An imbalance between excitation and inhibition, e.g., by disturbances in neuronal excitability or neurotransmitter systems and aberrant connectivity, underlies the generation of seizures (Goldberg and Coulter, 2013). Seizure incidence is higher in the neonatal period as compared to mature stages (Chapman et al., 2012) and seizures occurring in the neonatal period increase the risk of manifesting epilepsy (Pisani et al., 2015). Here we do not cover the mechanisms underlying the enhanced seizure incidence in the immature brain and refer to reviews by Sanchez and Jensen (2001) and Ben-Ari (2006).

The massively enhanced neuronal activity during a seizure causes a functional remodeling of neuronal circuits, which may increase the risk to develop further seizures, a concept termed “seizures beget seizures”. While this concept is still under discussion in the adult brain, experimental data demonstrate that neonatal seizures indeed increase the risk to manifest epilepsy (for review Ben-Ari and Holmes, 2006).

The most striking evidence comes from *in vitro* experiments with *in-toto* hippocampal preparations, which demonstrated that seizure-like activity in one hemisphere induces functional alterations in the contralateral hemisphere thereby inducing an epileptogenic mirror focus in rats (Khalilov et al., 2003). The induction of repetitive seizures during the first or second postnatal week enhances mossy fiber sprouting and the number granule cells in the rat hippocampus (Holmes et al., 1998) and persistently reduces spike adaptation, thus enhancing spike frequency (Villeneuve et al., 2000). Spontaneous febrile seizures in the immature rodent brain (at P10) have been shown to induce persistent changes in HCN channels (Chen et al., 2001). While repetitive seizures in rats induced during the first postnatal day reduce the amplitude of GABAergic postsynaptic currents (Isaeva et al., 2006), spontaneous febrile seizures enhance functional GABAergic projections in the hippocampus (Zhang et al., 2004a) and increase the expression of the GABA_A_ receptor α1 subunit (Zhang et al., 2004b). The developmental shift in the expression of chloride transporters, responsible for the shift from depolarizing to hyperpolarizing GABAergic action (Ben-Ari et al., 2012), is not affected by repetitive neonatal seizures (Isaeva et al., 2006). In accordance with these hippocampal results, repetitive seizures during the first 10 postnatal days also enhance the seizure susceptibility in the rat neocortex, which is accompanied by a small reduction in GABAergic postsynaptic events and a small increase in glutamatergic synaptic transmission (Isaeva et al., 2010). In summary, these observations suggest that altered neuronal activity during early developmental stages, e.g., caused by mutations in relevant genes or pre-/perinatal insults, may contribute to the establishment of certain forms of epilepsy, beside other mechanisms (Rakhade and Jensen, 2009).

One intriguing consequence of these observations is that it might be possible to prevent epileptogenesis after neonatal seizures or other early peri-/postnatal insults. A study in a genetic mouse model for neonatal epilepsies demonstrated that epileptiform-like activity and behavioral deficits in adult animals can be prevented by continuous application of the loop diuretics bumetanide, which reduces the depolarizing GABAergic responses and attenuates pathophysiological electrical activity (Ben-Ari et al., 2012) during the first two postnatal weeks (Marguet et al., 2015). However, clinical studies are less promising (Pressler et al., 2015).

### Schizophrenia

Schizophrenia is a relatively frequent and complex brain disorder. The risk of manifesting schizophrenia is inheritable in more than 50% of clinical cases (Schwab and Wildenauer, 2013), with environmental factors being an additional determinant. Schizophrenia is often considered as a neurodevelopmental disease, in which the combination of a genetic predisposition with insults during fetal, perinatal or early postnatal stages is required to manifest clinical symptoms (Lewis and Levitt, 2002). Such deleterious insults could be bacterial/viral infection and/or perinatal asphyxia during perinatal stages (Hagberg and Mallard, 2005). Experiments mimicking viral (Hartung et al., 2016) or bacterial (Nimmervoll et al., 2013) infections revealed changes in spontaneous activity patterns and hypoxia-ischemia also alters spontaneous activity in the immature rodent and human brain (Roberts et al., 2014; Ranasinghe et al., 2015; Mordel et al., 2016). Thus disturbances of neuronal activity under these conditions should be considered as an additional possible factor in the etiology of schizophrenia, beside other proposed mechanisms (Lisman et al., 2008). Interestingly, recent results indicate that the expression of the schizophrenia associated gene TCF4, a transcription factor implicated in neuronal development and plasticity, is tightly regulated by neuronal activity and that a point-mutation of this gene found in schizophrenic patients enhances its activity-dependent up-regulation (Sepp et al., 2017).

Recent experiments using mice that express a genetic background found in schizophrenic patients in combination with an intra-uterine insult, in this case a mimeticum for a viral infection, demonstrate that properties of spontaneous activity in the mouse prefrontal cortex changes already at early postnatal stages (Hartung et al., 2016). In line with these animal studies, cell cultures derived from induced pluripotent stem cells of schizophrenic patients exhibit a significant decrease in neuronal connectivity, decreased glutamate receptor expression and altered gene expression (Brennand et al., 2011; Roussos et al., 2016), suggesting that disturbed neuronal activity is an inherent feature of schizophrenia associated mutations. However, whether the altered activity during development only reflects disturbed neuronal connectivity caused by conditions that are associated with schizophrenia or contribute causally to the establishment of the disturbed functional connectivity in schizophrenic brains remains an open question.

### Autism Spectrum Disorders

ASD describe a heterogeneous class of disorders associated with social disability, communication impairment, repetitive behaviors and restricted interests (for review in Ecker et al., 2013). The etiology of ASD is complex and enigmatic, but a genetic background as well as environmental insults during early development are discussed (Geschwind and Levitt, 2007). The manifestation of ASD has been linked to prenatal insults such as maternal infections, maternal hormone status or influence of toxins and vitamins, as well as early postnatal insults, like maladaptive caregiver–infant interactions (Mandy and Lai, 2016). Is has been proposed that the complex trajectory of events leading from initial dysfunction to the manifestation of ASD initially involves molecular/cellular alterations causing functional changes (which in-turn alter molecular/cellular properties), suggesting that inappropriate neuronal activity may play an essential role during this process (Ecker et al., 2013).

It has been recently demonstrated that the frequency of GDPs is reduced in the immature hippocampus of BTBR-mice, which represents an animal model of idiopathic autism (Cellot et al., 2016). The same group demonstrated that expression of an ASD-related mutation of the postsynaptic adhesion molecule neuroligin-3 leads to an enhanced frequency of GDPs in the immature hippocampus (Pizzarelli and Cherubini, 2013). Knockout of neuroligin-4, another ASD related member of the neuroligin family, enhances the occurrence and duration of spontaneous spindle bursts in mouse cortex (Unichenko et al., 2017). In summary, these studies illustrate that an adequate amount of spontaneous activity may be the prerequisite for proper neurodevelopment, and both enhanced and reduced activity levels may lead to adverse outcomes.

One unequivocally identified cause for ASD is prenatal exposure of the anticonvulsant valproate (for review Roullet et al., 2013), which again indicates that altered neuronal activity should be considered as a possible factor in the etiology of ASD. Polymorphisms in genes encoding α_2_-subunit of glycine receptors have also been found in patients affected by ASD (Piton et al., 2011). Since glycine receptors do not considerably contribute to inhibition in the juvenile/mature cortex, this observation suggests that alterations in the glycinergic control of immature neuronal activity (Sava et al., 2014) and/or neuronal migration (Nimmervoll et al., 2011) may contribute to the manifestation of ASD in patients with this mutation. Finally, it is reported that brains from ASD patients often exhibit an excess number of neurons (Courchesne et al., 2011) and that in mice overproduction of cortical neurons causes autism-like disorders (Fang et al., 2014), indicating that enhanced neurogenesis (see “Neurogenesis” section) and/or impaired neuronal apoptosis (see “Neuronal Migration” section) also contribute to the etiology of ASD. In summary, these experimental and clinical findings provide some evidences that disturbances in spontaneous neuronal activity during early developmental stages might be an element that contributes to the etiology of ASD. However, more stringent electrophysiological experiments during early and late postnatal stages are required to reveal whether neuronal activity is a possible decisive factor, which link the diverse genomic and environmental causes to the specific neurophathological symptoms of ASD.

## Summary and Conclusion

In this review we provide a body of evidence indicating that spontaneous neuronal activity during early developmental stages is critically involved in a variety of neurodevelopmental processes. Altered neuronal activity seems to be involved in the etiology of neurological and neuropsychiatric diseases. Since the impact of genetic models on spontaneous neuronal activity has only occasionally been monitored, it is possible that several genetic models of neurodevelopmental diseases underestimate the contribution of electrical activity to the etiology of these disorders. In addition, we like to emphasize that our knowledge is rather limited, whether the specific spontaneous activity patterns occurring in a defined spatiotemporal sequence mediate distinct developmental processes. Further experiments, using a combination of electrophysiological, optogenetic and molecular techniques, will be required to address this intriguing question. However, even our limited knowledge on the impact of neuronal activity on early brain development already initiated new therapeutic options for the treatment of neurological and neuropsychiatric diseases and instructed that neurodevelopmental consequences of early pharmacological interventions must be considered.

## Author Contributions

SK, AS, OB, J-WY, HJL and WK wrote and corrected the manuscript.

## Conflict of Interest Statement

The authors declare that the research was conducted in the absence of any commercial or financial relationships that could be construed as a potential conflict of interest.
